# Socioeconomic Disparities in Multiple Myeloma Survival in New South Wales Australia: A Population-Based Cohort Study

**DOI:** 10.1177/10732748261438543

**Published:** 2026-04-24

**Authors:** Xue Qin Yu, Haoyu Zhang, Qingwei Luo, Anna Kelly, Marianne Weber, David Goldsbury, Karen Canfell, Eleonora Feletto

**Affiliations:** 1 The Daffodil Centre, 4334 The University of Sydney, and Cancer Council NSW, Sydney, NSW, Australia; 2 School of Public Health, 4334 The University of Sydney, Sydney, NSW, Australia; 3 Cancer Elimination Collaboration, School of Public Health, 4334 The University of Sydney, Sydney, NSW, Australia

**Keywords:** multiple myeloma, socioeconomic disparity, survival analysis, Australia

## Abstract

**Introduction:**

Australia has one of the highest incidence rates of multiple myeloma (MM) globally, and this burden is projected to increase significantly in the coming decades. Survival has improved over time, but it is not clear how this differs by socioeconomic group. Here, we used population-based data to evaluate survival differences by socioeconomic group and other prognostic factors of individuals with MM in Australia.

**Methods:**

This retrospective study included individuals diagnosed with primary MM between 2008 and 2019, as recorded in the New South Wales Cancer Registry, with survival follow-up to 2020. The identified individuals with primary MM were classified into 3 socioeconomic groups (low, medium, high) based on their residential location at diagnosis. Competing-risk modelling was used to estimate sub-hazard ratios (SHR) for socioeconomic group adjusting for potential prognostic factors, including age at diagnosis, sex, year of diagnosis, remoteness areas, autologous stem cell transplantation (ASCT) use, and hospital type.

**Results:**

Overall, 6,030 individuals were included in the study. The 5-year cumulative incidence of death due to MM was higher (p<0.0001) in low and medium socioeconomic groups (0.42 and 0.39), compared with the high socioeconomic group (0.34). Individuals in the high socioeconomic group were more likely to receive ASCT and to receive care at public principal referral/private hospitals. Compared to the high socioeconomic group, the excess risk of dying was higher (p<0.0001) in low (SHR=1.27, 95% CI: 1.14-1.42) and medium (SHR=1.20, 95% CI: 1.08-1.33) socioeconomic groups, but not statistically different (p=0.13) when other prognostic factors were considered.

**Conclusion:**

Survival disparity by socioeconomic groups among individuals with MM in Australia is largely accounted for by known prognostic factors, especially ASCT receipt and hospital type. Existing disparities suggest that a comprehensive evaluation of access to and availability of MM treatment, including identification of potential barriers to treatment receipt, is urgently needed.

## Introduction

Multiple myeloma (MM) is a type of blood cancer characterised by the proliferation of bone marrow plasma cells. The incidence of MM varies worldwide with Australia having one of the highest incidence rates which is projected to increase to 2043.^1^ A recent Australian study found that survival of individuals with MM over time has improved in line with treatment advances, but this was less pronounced in older age groups.^2^ As an incurable malignancy with no systematic population-based approaches to early detection, the prognosis of an individual with MM is dependent on the access to and quality of treatment.^3^ MM treatment approaches have changed over time moving from conventional chemotherapy to autologous stem cell transplantation (ASCT) from the 1990s to novel agents (proteasome inhibitor and immunomodulatory drugs),^4,5^ and now to the introduction of monoclonal antibodies and histone deacetylase (HDAC) inhibitors.^6^ Although HDAC inhibitors represented an earlier generation of novel therapies, the field has rapidly evolved, with bispecific antibodies, CART cell therapies, and antibody–drug conjugates now forming the most impactful innovations.^7^ Improved survival since mid-1990 has been attributed to these treatment changes and has resulted in longer duration of active treatment.^6,8^

The survival benefits of treatment advances may not be distributed uniformly across population subgroups such as for individuals from different socioeconomic groups, a noted influencing factor for MM and other cancer types.^6,9-11^ Socioeconomic disparities can lead to a wide difference in outcomes between population groups, which can occur, for example, when newly developed effective drugs are initially introduced into clinical practice.^12^ For MM, studies have reported less pronounced improvements in survival in those of poorer socioeconomic status (SES),^10,13-17^ though evidence in Australia is limited.^6,11,18^ Where available, early evidence suggested that for those diagnosed in the 1970s and 1980s, overall survival was consistent between socioeconomic groups.^18^ Although some later Australian studies found lower survival in people with MM from disadvantaged areas,^6,11^ there were no consistent disparities globally.^19^ The shift in available treatment and other characteristics may impact survival by socioeconomic groups differently.

In this study, we used linked-population-based data to evaluate survival differences by socioeconomic group and other prognostic factors of individuals with MM in Australia to highlight any disparities that might exist, and to assess whether key prognostic factors account for the survival disparities between the socioeconomic groups. Our underlying hypothesis was that SES influences MM survival through structural and health system–related mechanisms, including differences in access to optimal therapy, supportive care, and referral for procedures such as ASCT.

## Methods

Records of New South Wales (NSW) residents diagnosed with MM (ICD-O-3 morphology code 973) were obtained from the NSW Cancer Registry (NSWCR) through the Cancer Institute NSW’s Enduring Cancer Data Linkage (CanDLe). The current study cohort included individuals with a diagnosis of MM during the years 2008–2019 with mortality follow-up to December 2020, allowing a minimum of 1-year of follow-up. Notification of cancer diagnosis in NSW is a statutory requirement. NSW is Australia’s most populous state, encompassing highly urbanised metropolitan areas as well as extensive regional and remote communities. Australia’s universal healthcare system (Medicare) provides comprehensive coverage for hospital, medical, and pharmaceutical services. However, the state exhibits substantial socioeconomic and geographic variation, with disparities in health status and service access across levels of socioeconomic disadvantage and remoteness.^20^

The individuals diagnosed with MM were followed from the date of cancer diagnosis until the earliest of the following events by linking NSWCR data with cause of death records (the NSW Cause of Death Unit Record File) up to 31 December 2020: death due to MM-related causes or other causes, at 5-years after diagnosis, or end of the study period (31 December 2020). Individuals first diagnosed at death were excluded, along with very young (<20 years) or old individuals (≥90 years).

The outcome measure of this study is cause-specific survival after diagnosis of MM. In addition to the underlying cause of death from MM, we also include MM-related causes of death such as non-Hodgkin lymphoma or acute myeloid leukemia and potential MM therapy-related causes of death such as infections or renal failure to minimise possible misclassification.^21,22^ A sensitivity analysis, using the underlying cause of death from MM only as the outcome measure, was conducted to assess the robustness of the primary findings.

The study factor of interest was an individual’s SES according to an area-based measure at the time of diagnosis, using the index of relative socioeconomic disadvantage (IRSD) from the Australian Bureau of Statistics (ABS) census.^23^ The IRSD measures low income, low education attainment, low-skilled occupations and other aspects that may indicate a disadvantaged SES on a household basis.^23^ The three socioeconomic groups were categorised as low (three most disadvantaged deciles), medium (middle four deciles) and high (three least disadvantaged deciles).

### Covariates

In relation to treatment, ASCT was the only treatment type with comprehensive data available. ASCT status was ascertained when cancer registry records were linked with the NSW Admitted Patient Data Collection, which records all episodes of care from all public hospitals, multi-purpose services, private hospitals, and private day procedure centres in NSW. The ICD procedure code in the hospital records (13706-08, 13706-07 or 13760-00) were used to identify ASCT use.^24^ Other significant prognostic factors in univariate analyses were also included in the multivariable analysis including year of MM diagnosis, remoteness areas based on where the patient lived at diagnosis using the Accessibility/Remoteness Index of Australia Plus (ARIA+),^25^ and hospital type. Hospital type is ascertained by a person’s latest episode of care recorded in the hospital records, grouped as either public principal referral/private hospital or other public hospital and is a proxy measure for access and availability of treatment. A public principal referral hospital is indicative of a facility where there are a broad range of services available with specialist functions that are not always found in other public hospitals.^26^

### Statistical Analysis

Individual characteristics were summarized using descriptive statistics with frequency counts and percentages for categorical variables. The difference between individuals across socioeconomic groups was tested through the chi-squared test. Variables with non-significant differences between socioeconomic groups in univariate analysis were excluded (except for sex) from multivariable analysis.

Cancer-specific survival, or deaths attributed to MM, were estimated by the cumulative incidence function, treating deaths from all other causes as the competing risk. The Fine-Gray model^27^ was used to test associations between individual characteristics and mortality due to MM. Cox proportional hazards regression was used for fitting subdistribution hazard models to adjust for other prognostic factors.^28,29^ To quantify the extent to which the prognostic factors contributed to socioeconomic survival disparity, we fit three Cox models sequentially.^30^ The first model included socioeconomic group only, and the second model also adjusted for sex and was stratified by age group at diagnosis. The final model included socioeconomic group and other factors such as ASCT use,^4^ year of diagnosis,^2^ remoteness (ARIA+)^6^ and hospital type,^31^ with age group at diagnosis treated as a strata variable. ASCT was conceptualized as a mediator of the association between SES and MM survival, rather than a confounder. By comparing models without ASCT to model including ASCT we try to explore the degree to which SES differences in survival might operate through differential use of ASCT, recognizing that such adjustment blocks a major mediating pathway. Changes in the hazard ratio estimate when different combination of covariates were added served as model selection criteria. The changes were used to assess the extent of the variation in survival attributable to prognostic factors by socioeconomic group.

To check whether cross-border treatment might affect our results, we repeated the analysis after removing cases from five Local Health Districts near the NSW border.^32^ These patients may have received ASCT in other states, which would not appear in NSW records. Proportional hazard assumption for all variables was assessed by including an interaction term of a covariate and follow up time in the model.^33^ Age group was treated as a strata variable in the model due to violation of the proportionality assumption. No violation of the proportional hazard assumption was detected for other variables.

As this was a population-based study including all eligible individuals recorded in the registry during the study period, a formal sample size calculation was not performed. Given the statistical power resulting from the large sample size, a stringent level of significance (p<0.01) was used.^34^ All analyses were done using SAS version 9.4.

This analysis was covered by ethics approval for the CanDLe initiative from the NSW Population and Health Services Research Ethics Committee (HREC Approval 2019/ETH12584). All patient details have been de-identified and data were stored in the Secure Unified Research Environment (SURE) facility, a remote access computing environment to which authorised researchers were given encrypted access with strong authentication security. This study was conducted in accordance with the Helsinki Declaration of 1975, as revised in 2024,^35^ and its reporting conforms to the STROBE guidelines.^36^

## Results

A total of 6,030 individuals aged 20-89 years diagnosed with MM between 2008 and 2019 were included in the study. The median age at diagnosis of the cohort was 70 years with an interquartile range of 62-78 years, and 59.4% were males. There were significant differences in age distribution by socioeconomic group, with the high socioeconomic group having a greater proportion of people in the younger age group (20-69), 50% vs 43% for low socioeconomic group (Table 1). In addition, more individuals (85.8%) from the high socioeconomic group lived in major cities at the time of diagnosis than from other groups (56.3%-65.3%), and they were more likely to receive ASCT and to be cared at public principal referral/private hospitals (Table 1). In contrast, there were no significant differences in sex and region of birth across three socioeconomic groups.

**Table 1.** Characteristics of the Study Population: Individuals Diagnosed With Multiple Myeloma During 2008-2019 in NSW, Australia by Socioeconomic Group

​	Socioeconomic group			p-value^†^
	Low (n=1873)	Medium (n=2454)	High (n=1703)	
Age group (years)	​	​	​	<0.0001
20-69	806 (43.0%)	1208 (49.2%)	852 (50.0%)	​
70-79	606 (32.3%)	722 (29.4%)	518 (30.4%)	​
80-89	461 (24.6%)	524 (21.4%)	333 (19.6%)	​
Sex	​	​	​	0.33
Male	1087 (58.0%)	1466 (59.7%)	1028 (60.4%)	​
Female	786 (42.0%)	988 (40.3%)	675 (39.6%)	​
Region of birth	​	​	​	0.038
ANZ-born	1286 (68.7%)	1670 (68.1%)	1120 (65.8%)	​
European-born	337 (18.0%)	499 (20.3%)	354 (20.8%)	​
Asian-born	102 (5.4%)	140 (5.7%)	109 (6.4%)	​
Born elsewhere	148 (7.9%)	145 (5.9%)	120 (7.0%)	​
Remoteness area	​	​	​	<0.0001
Major cities	1055 (56.3%)	1603 (65.3%)	1461 (85.8%)	​
Inner regional	555 (29.6%)	656 (26.7%)	224 (13.2%)	​
Other areas^*^	263 (14.0%)	195 (7.9%)	18 (1.1%)	​
ASCT use	​	​	​	<0.0001
Yes	327 (17.5%)	585 (23.8%)	464 (27.3%)	​
No	1546 (82.5%)	1869 (76.2%)	1239 (72.8%)	​
Hospital type	​	​	​	<0.0001
Public principal referral/private	1114 (59.5%)	1723 (70.2%)	1485 (87.2%)	​
Other public	759 (40.5%)	731 (29.8%)	218 (12.8%)	​

ANZ: Australia or New Zealand; ASCT: autologous stem cell transplant.

^*^Including outer regional, remote and very remote areas.

^†^P-value for Chi-squared test.

**Figure 1.**
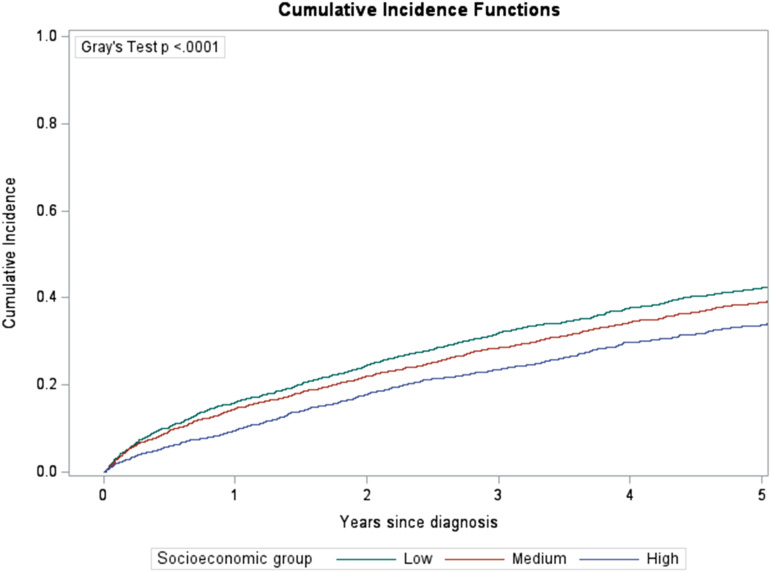
Cumulative incidence of deaths due to multiple myeloma by socioeconomic group

**Figure 2.**
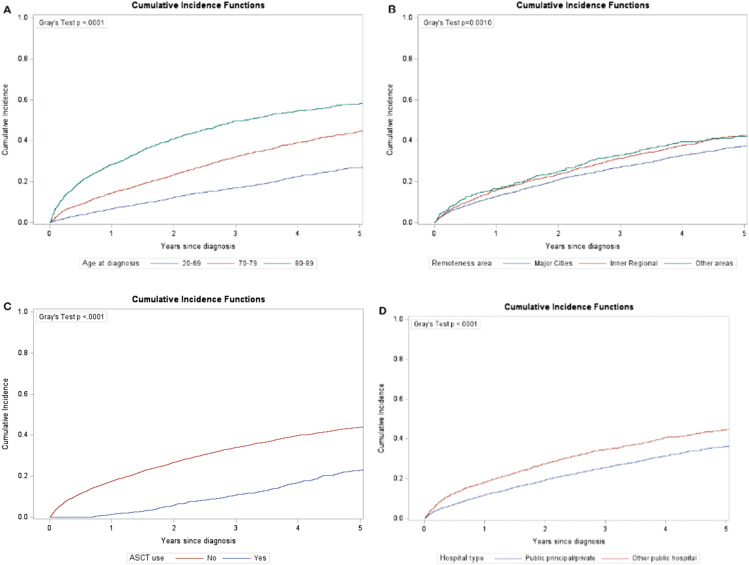
Cumulative incidence of deaths due to multiple myeloma by age at diagnosis (A), remoteness areas (B), ASCT use (C) and hospital type (D)

Cumulative incidence of deaths attributable to MM by socioeconomic group are presented in Figure 1. Cumulative incidence varied significantly by socioeconomic group (Gray’s test p<0.0001), with 5-year cumulative incidence being higher for those living in low (0.42, 95% confidence interval (CI): 0.40-0.45) and medium (0.39, 95% CI: 0.37-0.41) socioeconomic areas than those in high (0.34, 95% CI: 0.31-0.36) socioeconomic areas. Similarly, significant variations were observed in other prognostic factors (Gray’s test p<=0.001) including age at diagnosis, remoteness, use of ASCT and hospital type (Figure 2). The largest variation was seen between age group at diagnosis (20-69:0.27, 70-79:0.45, 80-89:0.58) and ASCT use (Yes: 0.23 vs No: 0.45). Values of these cumulative incidence estimates, and their 95% CI are presented in the Supplemental Table 1.

### Multivariable Analyses

The estimated sub-hazard ratios (SHRs) by socioeconomic group are reported in Table 2. In the univariable regression (model 0), compared to those in the high socioeconomic group, the hazard of deaths due to MM were significantly higher for individuals living in low (SHR=1.27) and medium (SHR=1.20) socioeconomic areas (p< 0.0001). With minimal adjustment (sex and age group), the excess risk of dying was reduced slightly (SHR = 1.23, 1.19 for low and medium socioeconomic groups, respectively) but remained significant (p=0.0005) (model 1 in Table 2). Upon further adjusting for additional prognostic factors (model 2 in Table 2) including year of diagnosis, remoteness (ARIA+), ASCT use, and hospital type, the socioeconomic disparities in survival became statistically insignificant (p=0.13).

**Table 2.** Sub-Hazard Ratios (SHR) From Subdistribution Hazard Models for Death Due to Multiple Myeloma by Socioeconomic Groups in New South Wales, Australia

Socioeconomic groups (SES)	SHR and 95% confidence interval					
	Model 0		Model 1		Model 2	
High	1.00	(Reference)	1.00	(Reference)	1.00	(Reference)
Medium	1.20	(1.08-1.33)	1.19	(1.07-1.33)	1.10	(0.98-1.22)
Low	1.27	(1.14-1.42)	1.23	(1.10-1.38)	1.12	(.997-1.26)
p-value for SES^†^	<0.0001		0.0005		0.13	

*Note.* Model 0 includes SES only; Model 1 includes SES, sex, stratified by age group at diagnosis; Model 2 includes SES, sex, and other prognostic factors (year of diagnosis, remoteness (ARIA+), ASCT use, and hospital type) stratified by age group.

^†^p-value for the effect of socioeconomic groups in the Cox model.

A sensitivity analysis showed very similar results for MM only as the underlying cause of death, confirming the robustness of the primary findings (Supplemental Table 2). Excluding five NSW border Local Health Districts to reduce cross-border care effects also did not materially alter the results (Supplemental Tables 3 and 4).

## Discussion

Based on our univariate analysis of over 6000 individuals with MM in NSW, the 5-year cumulative incidence of death due to MM was lowest in the high socioeconomic group,^10^ the younger age group,^2^ those in major cities,^11^ those who received ASCT,^4,37^ and those who received care in a public principal referral or private hospital.^31^ The excess risk of dying was higher in medium and low socioeconomic groups, even after accounting for age and sex, but was no longer statistically different when other prognostic factors were considered. Our findings reinforce previously reported MM survival disparities in the univariable analysis, aligning with reported higher survival in younger people as well as for people living in major cities and for people who received ASCT.^2,37,38^

In the multivariable analysis, we sequentially tested each model including various prognostic factors. This way we could examine to what extent they explain the survival variations attributable to socioeconomic group. Some variables, such as ASCT use and remoteness, despite being correlated with socioeconomic group, provided independent predictive value as indicated in Model 2. Despite being reported as a key prognostic factor for MM survival,^10^ socioeconomic group was not a significant predictor of MM-specific death when other prognostic factors were included in the analysis. The multivariable Cox Model 2 presented the combination of prognostic factors that yielded less survival variations between socioeconomic group but was not significant. This suggests that socioeconomic disparities are largely accounted for by factors related to year of diagnosis, remoteness, ASCT utilisation, and hospital type.

SES has been associated with access and utilisation of treatment for cancer.^9,19,37,39,40^ In Australia, Ai et al (2024) found that lower SES was associated with a reduction in ASCT utilisation and lower survival.^37^ Although ASCT and other key treatments for MM, such as bortezomib, have been subsidised through Australia’s universal health care system,^41^ access to treatment, and their utilisation may not be consistent across socioeconomic groups. Data on treatments other than ASCT were not available to explore whether this effect extends to other treatments. Disparities based on remoteness were also highlighted in our study. Individuals with MM living in major cities had better survival but after adjusting for other prognostic factors, location did not significantly influence the excess risk of dying from MM. As a result, the costs and location cannot be associated with the disparities in survival directly. As people in Australia generally travel to metropolitan and regional hubs for MM-related treatment,^37^ the differential survival in socioeconomic groups may be partially attributed to the indirect and out-of-pocket expenses, including travel and non-remunerated leave incurred as part of ongoing treatment and care, but there is no reported MM-specific data on this topic.

Our finding that individuals with MM who received care in public principal referral or private hospitals had lower MM death rate than those treated in other public hospitals was in line with previous studies. A recent Australian study found that individuals with MM treated in a rural hospital (other public hospital category) had shorter survival than those treated at a public principal referral hospital.^31,37^ A systematic review of 10 studies including 85,198 people with MM from multiple countries suggested that the case volume of treating facilities was a prognostic factor. The study found that facilities with low case volumes had higher MM mortality rates than those with high volume.^10^ Differential outcomes by facility may be due in part to variability in resource access and availability as well as healthcare provider experience. Generally, facilities with high case volumes are more likely to offer a wider range of treatment options and more opportunities to participate in clinical trials and provide multidisciplinary care,^31^ all of which may contribute to better outcomes.

A key strength of this study is the large sample size which is not always available for less common cancer types such as MM. While the data are from one region in Australia, NSW represents one third of the Australian population and is largely generalisable to the overall MM population, with MM incidence and mortality rates are almost identical to the national rates.^42^ Given the limited curative rate of MM, we used cumulative incidence functions to account for competing risks of death. This approach is preferable to conventional survival analysis because it inherently reduces residual confounding arising from SES-related differences in competing mortality risks.^43^ Additionally, we addressed the MM-related causes of death and have allowed for this in our analysis to ensure the survival disparities are not underestimated. The results aligned with the supplementary analysis of MM-specific deaths thus providing robust estimates of MM survival by socioeconomic group.

Our study is not without its limitations. Due to data availability, we were unable to ascertain the full treatment history for MM; only information on ASCT utilisation was available and included in the analysis. Genomic data were similarly unavailable because of these constraints. Finally, the measure of socioeconomic group derived from the ABS census used an area-based measure at the time of diagnosis. This measure may not accurately capture an individual’s current socioeconomic status, which could introduce misclassification bias.

## Conclusion

In this population-based study of individuals with MM in NSW, we found that disparities in survival across socioeconomic groups were largely explained by differences in ASCT treatment and hospital type. These findings highlight the critical importance of equitable access to ASCT and to private or principal referral hospitals, which could significantly improve outcomes for individuals from lower socioeconomic groups. A comprehensive evaluation of access to and availability of MM treatment, including identification of potential barriers to treatment receipt, is urgently needed.

## Supplemental Material

Supplemental Material - Socioeconomic Disparities in Multiple Myeloma Survival in New South Wales Australia: A Population-Based Cohort Study Supplemental Material for Socioeconomic Disparities in Multiple Myeloma Survival in New South Wales Australia: A Population-Based Cohort Study by Xue Qin Yu, Haoyu Zhang, Qingwei Luo, Anna Kelly, Marianne Weber, David Goldsbury, Karen Canfell and Eleonora Feletto in Cancer Control.

## Appendix

### Abbreviations

MM: Multiple myeloma
ASCT: Autologous stem cell transplantation
SHR: Sub-hazard ratios
HDAC: Histone deacetylase
SES: Socioeconomic status
NSW: New South Wales
NSWCR: NSW Cancer Registry
CanDLe: Enduring Cancer Data Linkage
IRSD: Index of relative socioeconomic disadvantage
ABS: Australian Bureau of Statistics
ARIA+: Accessibility/Remoteness Index of Australia Plus
CI: Confidence interval
ANZ: Australia or New Zealand
