# Supplementary material for: Socioeconomic Disparities in Multiple Myeloma Survival in New South Wales Australia: A Population-Based Cohort Study
Source: Cancer Control. 2026 Apr 24;33:10732748261438543. doi: 10.1177/10732748261438543 (PMC13125840; doi:10.1177/10732748261438543)
Supplement: Supplemental Material - Socioeconomic Disparities in Multiple Myeloma Survival in New South Wales Australia: A Population-Based Cohort Study [file sj-pdf-1-ccx-10.1177_10732748261438543.pdf]

**Socioeconomic disparities in multiple myeloma survival in New South Wales Australia**

## Supplementary Materials

**Sup Table 1. 5-year cumulative incidence of death due to multiple myeloma by main prognostic factors, NSW, Australia 2008-2019**

| | Cumulative incidence | 95% confidence intervals | p-value† |
|---|---|---|---|
| **Socioeconomic group** | | | <0.0001 |
| Low | 0.43 | (0.41-0.45) | |
| Medium | 0.39 | (0.37-0.41) | |
| High | 0.34 | (0.32-0.37) | |
| **Age at diagnosis (year)** | | | <0.0001 |
| 20-69 | 0.27 | (0.25-0.29) | |
| 70-79 | 0.45 | (0.42-0.47) | |
| 80-89 | 0.58 | (0.55-0.61) | |
| **Remoteness areas** | | | 0.001 |
| Major cities | 0.37 | (0.36-0.39) | |
| Inner regional | 0.43 | (0.40-0.45) | |
| Other areas | 0.42 | (0.37-0.47) | |
| **ASCT‡ use** | | | <0.0001 |
| Yes | 0.23 | (0.20-0.25) | |
| No | 0.44 | (0.42-0.46) | |
| **Hospital type** | | | <0.0001 |
| Public principal/private | 0.37 | (0.35-0.38) | |
| Other public | 0.45 | (0.42-0.47) | |

† Gray's test.
‡ Stands for autologous stem cell transplantation.

## Results from sensitivity analysis

**Sup Table 2. Sub-hazard ratios (SHR) from subdistribution hazard models for death due to multiple myeloma (underlying cause only) by socioeconomic groups in New South Wales, Australia**

| Socioeconomic groups (SES) | SHR and 95% confidence interval | | | | | |
|---|---|---|---|---|---|---|
| | Model 0 | | Model 1 | | Model 2 | |
| High | 1.00 | | 1.00 | | 1.00 | |
| Medium | 1.24 | (1.11-1.38) | 1.23 | (1.10-1.37) | 1.14 | (1.02-1.27) |
| Low | 1.31 | (1.17-1.47) | 1.28 | (1.14-1.43) | 1.17 | (1.03-1.31) |
| p-value for SES† | <0.0001 | | <0.0001 | | 0.03 | |

Note: Model 0 includes SES only; Model 1 includes SES, sex, stratified by age group at diagnosis; Model 2 includes SES, sex, and treatment-related factors (year of diagnosis, remoteness, ASCT use, and hospital type), stratified by age group,

† p-value for the effect of socioeconomic groups in the Cox regression model.

## Results from excluding cases from five local health districts near the NSW border

**Sup Table 3. 5-year cumulative incidence of death due to multiple myeloma by socioeconomic status and ASCT‡ use, NSW, Australia 2008-2019**

| | Cumulative incidence | 95% confidence intervals | p-value† |
|---|---|---|---|
| **Socioeconomic group** | | | <0.0001 |
| Low | 0.43 | (0.40-0.45) | |
| Medium | 0.40 | (0.38-0.42) | |
| High | 0.35 | (0.32-0.37) | |
| **ASCT use** | | | <0.0001 |
| Yes | 0.23 | (0.21-0.26) | |
| No | 0.44 | (0.43-0.46) | |

† Gray's test.
‡ Stands for autologous stem cell transplantation.

**Sup Table 4. Sub-hazard ratios (SHR) from subdistribution hazard models for death due to multiple myeloma by socioeconomic groups in New South Wales, Australia**

| Socioeconomic groups (SES) | SHR and 95% confidence interval | | | | | |
|---|---|---|---|---|---|---|
| | Model 0 | | Model 1 | | Model 2 | |
| High | 1.00 | | 1.00 | | 1.00 | |
| Medium | 1.20 | (1.07-1.34) | 1.19 | (1.07-1.33) | 1.11 | (0.99-1.24) |
| Low | 1.23 | (1.09-1.38) | 1.20 | (1.06-1.35) | 1.10 | (0.97-1.24) |
| p-value for SES† | 0.0009 | | 0.0028 | | 0.18 | |

Note: Model 0 includes SES only; Model 1 includes SES, sex, stratified by age group at diagnosis; Model 2 includes SES, sex, and treatment-related factors (year of diagnosis, remoteness, ASCT use, and hospital type), stratified by age group,

† p-value for the effect of socioeconomic groups in the Cox regression model.
